# Mediation by differential DNA methylation of known associations between single nucleotide polymorphisms and bladder cancer risk

**DOI:** 10.1186/s12881-020-01172-1

**Published:** 2020-11-19

**Authors:** Kristina M. Jordahl, Amanda I. Phipps, Timothy W. Randolph, Lesley F. Tinker, Rami Nassir, Lifang Hou, Garnet L. Anderson, Karl T. Kelsey, Emily White, Parveen Bhatti

**Affiliations:** 1 Department of Epidemiology, School of Public Health, University of Washington, Box 357236, Seattle, WA 98195 USA; 2 Division of Public Health Sciences, Fred Hutchinson Cancer Research Center, Seattle, WA USA; 3 Department of Biochemistry and Molecular Medicine, University of California, Davis, CA USA; 4 Department of Preventive Medicine, Northwestern University Feinberg School of Medicine, Chicago, IL USA; 5 Departments of Epidemiology and Pathology and Laboratory Medicine, Brown University, Providence, RI USA; 6 Cancer Control Research, BC Cancer, Vancouver, BC Canada

**Keywords:** Bladder cancer risk SNPs, DNA methylation, Bladder cancer, Mediation analysis

## Abstract

**Background:**

Though bladder cancer has been the subject of many well-powered genome-wide association studies, the mechanisms involving bladder-cancer-associated single nucleotide polymorphisms (SNPs) remain largely unknown. This study focuses on rs798766, rs401681, rs2294008, and rs8102137, which have been associated with bladder cancer and are also *cis*-acting methylation quantitative loci (mQTL).

**Methods:**

Among 412 bladder cancer cases and 424 controls from the Women’s Health Initiative (WHI), we assessed whether the effects of these SNPs on bladder cancer are mediated through proximal DNA methylation changes in pre-diagnostic blood at mQTL-associated CpG sites, which we refer to as natural indirect effects (NIEs). We used a multiple-mediator mediation model for each of the four mQTL adjusted for matching variables and potential confounders, including race/ethnicity, smoking status, and pack-years of smoking.

**Results:**

While not statistically significant, our results suggest that substantial proportions of the modest effects of rs401681 (*OR*^*NIE*^ = 1.05, 95% confidence interval (CI) = 0.89 to 1.25; NIE percent = 98.5%) and rs2294008 (*OR*^*NIE*^ = 1.10, 95% CI = 0.90 to 1.33; NIE percent = 77.6%) on bladder cancer risk are mediated through differential DNA methylation at nearby mQTL-associated CpG sites. The suggestive results indicate that rs2294008 may affect bladder cancer risk through a set of genes in the lymphocyte antigen 6 family, which involves genes that bind to and modulate nicotinic acetylcholine receptors. There was no suggestive evidence supporting mediation for rs8102137 and rs798766.

**Conclusions:**

Though larger studies are necessary, the methylation changes associated with rs401681 and rs2294008 at mQTL-associated CpG sites may be relevant for bladder carcinogenesis, and this study demonstrates how multi-omic data can be integrated to help understand the downstream effects of genetics variants.

## Introduction

Bladder cancer has been the subject of multiple well-powered genome-wide association studies, which have identified a robust set of single nucleotide polymorphisms (SNPs) associated with bladder cancer risk [1]. As is the case for most complex diseases, the mechanisms underlying these associations remain largely unknown. Since changes in DNA methylation may reveal shifts in epigenetic state or in gene expression, we explored whether proximal changes in DNA methylation are causally involved in the effects of these SNPs on bladder cancer risk to improve our understanding of their carcinogenic downstream effects.

Our study focuses on rs798766, rs401681, rs2294008, and rs8102137. These SNPs have been associated with bladder cancer in genome-wide association studies and are also methylation quantitative loci (mQTL), since they affect patterns of DNA methylation at nearby CpG sites [2]. For rs2294008, we also follow up on preliminary evidence that the T risk allele has a stronger effect in smokers [3], where smoking is the most important known risk factor for bladder cancer.

Based on queries of the Accessible Resource for Integrated Epigenomic Studies (ARIES) mQTL database [4], 50% of the eight SNPs associated with bladder cancer were mQTL in blood, as compared to only 34% of total SNPs across the entire genome. This is consistent with genome-wide association results for other complex traits, which are often enriched for *cis* mQTL [4], suggesting that mQTL SNPs associated with complex diseases like bladder cancer may have effects that can be detected by differential DNA methylation. Our study formally investigates the potential mediation of the association between mQTL SNPs and bladder cancer risk through methylation changes in blood at mQTL-associated CpG sites. This study represents an important step toward integrating genetic and epigenetic data and improving our understanding of the mechanisms related to genetic variants involved in susceptibility to bladder cancer.

## Patients and methods

### Study participants

The current study involved participants from a case-control study of pre-diagnostic DNA methylation and bladder cancer, which was nested in the Women’s Health Initiative (WHI) [5]. Briefly, the 161,808 postmenopausal women in were recruited across the US from 1993 to 1998 in the clinical trials of hormone therapy, dietary modification, and calcium/vitamin D supplementation and in the observational study arms [6]. The WHI Extension Studies collected health outcomes information on consenting WHI participants after 2005.

The initial case-control study included 440 WHI participants diagnosed with transitional cell carcinoma of the bladder during follow-up and 440 controls without cancer. Cases and controls were matched on year of enrollment, age at enrollment, follow-up time, trial component, and DNA extraction method [5]. Subjects were excluded if they had missing race/ethnicity (*n* = 1) or pack-years information (*n* = 35), or if salt or Bioserve was used as the DNA extraction method (*n* = 8), leaving a sample size of 412 cases and 424 controls.

### Data and biospecimen collection

Age and race/ethnicity was self-reported during the screening process for the WHI [6]. The smoking information used in our analyses was collected through baseline questionnaires and used to determine smoking status and pack-years of smoking [7].

### DNA methylation data

Genome-wide DNA methylation was assessed based on fasting blood samples collected at baseline and stored as buffy coats at − 70 °C. Methylation M-values, which are the base-2 logit of the methylated signal divided by the total signal, were measured at each autosomal CpG site using the Illumina 450 K Infinium HumanMethylation Bead Array [5, 8]. During pre-processing and quality control, we applied background correction and functional normalization and excluded CpG sites based on probe detection, beadcount, proximity to common SNPs, and cross-reactivity [5].

### Identifying mQTL SNPs associated with bladder cancer

A meta-analysis of bladder cancer genome-wide association studies conducted in 2014 combined all previous studies to verify known loci and reported genome-wide significant associations (*p* < 5e-8) for eight SNPs: rs710521, rs798766, rs401681, rs1495741, rs2294008, rs9642880, rs8102137, and rs1014971 [1]. The meta-analysis included an average of 22 studies involving an average of 11,131 cases and 50,634 controls for each reported association. We then used the ARIES mQTL database to assess whether each of these eight SNPs was also an mQTL [4]. This publicly available dataset includes approximately 1000 mother-offspring pairs from the Avon Longitudinal Study of Parents and Children. Associations between each of 8,282,911 directly genotyped or imputed common SNPs and each of 395,625 CpG sites on the Illumina Infinium HumanMethylation450 Bead Array that passed QC are provided in the mQTL database. To most closely match the population of older women included in the WHI, we only evaluated the methylation data measured in peripheral blood samples collected from mothers at the middle age time point (mean age = 47.45; *n* = 742). For each SNP, we searched for associated CpG sites in the MatrixEQTL database and then restricted to SNP-CpG results with a *p*-value below 1e-14 to control the false positive rate at 0.2% [4]. We also restricted to CpG sites that passed our quality control steps, leaving four mQTL that were associated with methylation changes at a total of ten CpG sites. Notably, the risk allele is the minor allele for all selected SNPs except for rs401681.

### SNP genotyping

Taqman® SNP Genotyping Assays were used to determine the genotypes for each mQTL SNP. For the assay, 5 ng of genomic DNA was aliquoted into 384-well plates and dried down. Each assay was combined with Taqman® Genotyper master mix, run under universal PCR conditions, and analyzed with the ABI 7900 HT Taqman® Real-Time instrument.

Prior to running the study samples, 90 samples representing 30 parent-parent-child trios from a population of Utah residents with European ancestry (CEPH) were genotyped to assess performance of the genotyping assays. Assay accuracy was verified by comparing genotypes to publicly available genotype data for these samples from SNP500 Cancer [9] and dbSNP [10] and by assessing inheritance errors. As another quality control step, two external control samples from the HapMap project were included on each plate to confirm reliability and reproducibility of the genotyping across the study plates. Inter-plate duplicates were also included. Samples with weak signals and outliers were repeated once. There was 100% concordance among the duplicates. All SNPs had a greater than 99% call rate and were in Hardy-Weinberg equilibrium.

### Statistical analyses

Mediation analyses were conducted with each of the four mQTL (rs798766, rs401681, rs2294008, and rs8102137) as the exposure, all corresponding CpG sites as the mediators, and incident bladder cancer as the outcome. We estimated causal effects using a regression-based approach for dichotomous outcomes [11, 12], which is based on a counterfactual framework for causal inference. This method uses a logistic regression model for the outcome (1) and a linear regression model for the mediator (2), where *A* denotes the exposure, *M* denotes the mediator(s), and *Y* denotes the outcome of interest. *a* and *m* denote fixed levels of *A* and *M*, respectively. The logistic regression model was fit among cases and controls (*n* = 836) and the linear regression model was fit only among controls (*n* = 424).

1 $$ \mathrm{logit}\left(P\left(Y=1|a,m,c\right)\right)={\theta}_0+{\theta}_1a+{\sum}_{i=1}^K{\theta}_2^{(i)}{m}^{(i)}+{\theta}_4^{\prime }c $$

2 $$ E\left[{M}^{(i)}|a,c\right]={\beta}_0^{(i)}+{\beta}_1^{(i)}a+{\beta_2^{(i)}}^{\prime }c $$

for *i* = 1, …, *K* mediator(s). The models were adjusted for a vector of covariates, *c*, that included our matching variables and potential confounders. Specifically, we adjusted our analyses for WHI arm (hormone therapy/dietary modification, hormone therapy, dietary modification, observational study), age at baseline (continuous), year of enrollment (continuous: 1994–1995 = 1, 1996 = 2, 1997 = 3, 1998 = 4), follow-up time (continuous), DNA extraction method (5-prime, phenol), race/ethnicity (Asian/Pacific Islander, Black/African American, Hispanic, non-Hispanic White, other), smoking status (never, former, current), and pack-years of smoking (continuous).

Using the coefficients estimated from regression models (1) and (2), the following equations were used to estimate the odds ratio (*OR*) per risk allele for the natural direct effect (NDE) and the natural indirect effect (NIE) [11]:

$$ O{R}^{NDE}=\exp \left({\theta}_1\right) $$

$$ O{R}^{NIE}=\exp \left({\sum}_{i=1}^K{\beta}_1^{(i)}{\theta}_2^{(i)}\right) $$

Effect estimates and confidence intervals (CIs) were estimated using the bootstrapping approach based on 1000 bootstrap samples. The *OR*^*NDE*^ captures the effect of the exposure on the outcome that does not act through the CpG site mediators, while the *OR*^*NIE*^ captures the effect of the exposure on the outcome that acts through the CpG site mediators. The total effect (*OR*^*TE*^) is the product of the *OR*^*NDE*^ and *OR*^*NIE*^, although this relationship is not exact when using the bootstrapping approach. The method allows the effects of individual mediators to cancel one another, since each SNP affects its associated CpG sites simultaneously. The NIE percent is the percent of the total effect attributed to the natural indirect effect (*OR*^*NIE*^/*OR*^*TE*^).

The mediation analyses were conducted based on a macro developed by Valeri and VanderWeele [13] using SAS® software (versions 9.3 and 9.4, SAS® Institute Inc., Cary, NC, USA). The macro was modified to produce multiple-mediator effect estimates based on existing equations [11] and to provide bootstrap-based confidence intervals. The code for the multiple-mediator macro used for these analyses is available at: https://github.com/kristina-jordahl/vanderweele_mediation_analysis_with_multiple_mediators.

## Results

Table 1 provides the distribution of demographic and clinical characteristics by case-control status for our study population. Compared to controls, a larger proportion of cases were of White race/ethnicity, were past and current smokers, and had a heavier smoking history of at least 30 pack-years.

**Table 1:** Distribution of demographic and clinical characteristics among bladder cancer cases and controls from the Women's Health Initiative

	Cases (***n*** = 412)	Controls (***n*** = 424)
**WHI Arm (N, %)**		
OS	213 (52%)	218 (52%)
CT: HT and DM	24 (6%)	26 (6%)
CT: HT only	55 (13%)	56 (13%)
CT: DM only	120 (29%)	124 (29%)
**Age (N, %)**		
< 50–59	97 (24%)	100 (24%)
60–69	194 (47%)	203 (48%)
70–79+	121 (29%)	121 (28%)
**Year of Enrollment (N, %)**		
1994 - 1995	105 (25%)	109 (26%)
1996	125 (30%)	127 (30%)
1997	114 (28%)	116 (27%)
1998	68 (17%)	72 (17%)
**DNA Extraction Method (N, %)**		
5-prime	397 (96%)	408 (96%)
Phenol	15 (4%)	16 (4%)
**Race/Ethnicity (N, %)**		
Asian/Pacific Islander	4 (1%)	12 (3%)
Black/African American	24 (6%)	40 (9%)
Hispanic	7 (2%)	16 (4%)
Non-Hispanic White	375 (91%)	349 (82%)
Other	2 (< 1%)	7 (2%)
**Smoking (N, %)**		
Never Smoked	155 (38%)	234 (55%)
Past Smoker	204 (49%)	172 (41%)
Current Smoker	53 (13%)	18 (4%)
**Pack-Years (N, %)**		
Never Smoker	155 (38%)	234 (55%)
> 0 - < 30	145 (35%)	137 (32%)
≥ 30	112 (27%)	53 (13%)

Abbreviations: *OS* Observational study; *CT* Clinical trials; *HT* Hormone therapy clinical trial; *DM* Dietary modification clinical trial

For each mQTL, there was a range of one to five mQTL-associated CpG sites (Table 2). All of our selected mQTL were *cis*-acting, since they were within 1 Mb of their associated CpG sites. According to ARIES, these mQTL are common SNPs with risk allele frequencies ranging from 0.20 to 0.56 and are mostly located in or near CpG islands. These SNPs have effect sizes ranging from a 0.4 to 3% difference in median proportion methylated between homozygote groups. These SNPs are associated with an increase in methylation for three CpG sites and a decrease in methylation for seven CpG sites.

**Table 2:** Selected mQTL and their associated CpG sites

SNP	SNP Location	RA (RAF)	CpG	CpG Location	CpG Gene	CpG Gene Group^**a**^	Relation to CpG Island^**b**^	ARIES Direction^**c**^	ARIES Effect Size^**d**^
rs798766	chr4:1734239	T (0.23)	cg00006948	chr4:1768889			Island	+	0.004
rs401681	chr5:1322087	C (0.53)	cg27028750	chr5:1349422			S_Shelf	+	0.059
			cg26209169	chr5:1316264				–	0.038
rs2294008	chr8:143761931	T (0.49)	cg06565975	chr8:143823917	*SLURP1*	TSS200	S_Shelf	–	0.041
			cg03405983	chr8:143858548	*LYNX1*	5’UTR/TSS200/1stExon	Island	–	0.009
			cg24023258	chr8:143781297	*LY6K*	TSS1500	N_Shore	+	0.004
			cg17888033	chr8:143858414	*LYNX1*	5’UTR/1stExon	Island	–	0.020
			cg17252645	chr8:143867129	*LY6D*	Body		–	0.042
rs8102137	chr19:30296853	C (0.32)	cg16836589	chr19:30303674	*CCNE1*	1stExon/Body	Island	–	0.011
			cg27475126	chr19:30303651	*CCNE1*	1stExon/Body	Island	–	0.049

^a^Functional region of gene as indicated in v1.2 Illumina annotation: TSS1500 = 200–1500 bases upstream of the transcription start site; TSS200 = 0–200 bases upstream of the transcription start site; 5’UTR = 5 prime untranslated region; 1stExon = First segment of gene coding for peptide sequence; Body = Between ATG and stop codon; 3’UTR = Between stop codon and poly A signal; Multiple listings indicate locus is in a region with multiple splice variants

^b^Position relative to CpG island as indicated in v1.2 Illumina annotation: Island = CpG island (CG content > 50%, Obs/Exp CpG ratio > 0.60, and length > 200 bps); OpeanSea = Non-island region; Shore = 0–2 kb flanking CpG island; Shelf = 2–4 kb flanking CpG island

^c^Direction of association between mQTL and CpG site reported in the ARIES mQTL database

^d^Effect size (defined as the difference in median proportion methylated between homozygote groups) between mQTL and CpG site reported in the ARIES mQTL database

Abbreviations: *RA* Risk allele; *RAF* Risk allele frequency

The results from the mediation analyses for rs798766, rs401681, rs2294008, and rs8102137 are presented in Table 3. Only rs8102137 had a statistically significant total effect, and none of the natural indirect effects through differential methylation of mQTL-associated CpG sites were statistically significant. However, our results suggest that almost all (NIE percent = 98.5%) of the modest effect of rs401681 on bladder cancer risk (*OR*^*TE*^ = 1.05) acts through cg27028750 and cg26209169 (*OR*^*NIE*^ = 1.05, 95% CI = 0.89 to 1.25). Similarly, a large NIE proportion (NIE percent = 77.6%) suggests that the total effect of rs2294008 on bladder cancer risk (*OR*^*TE*^ = 1.13) may act through cg06565975, cg03405983, cg24023258, cg17888033, and cg17252645 (*OR*^*NIE*^ = 1.10, 95% CI = 0.90 to 1.33). There was little suggestive evidence to support mediation through changes in DNA methylation for the associations of rs8102137 and rs798766 with bladder cancer risk.

**Table 3:** Mediating effects of mQTL-associated CpG site(s) for association between each SNP and bladder cancer risk

SNP	Region	Gene	RA	Mediator(s)	Effect of SNP^a^	OR	95% CI
rs798766	4p16.3	*TMEM129-TACC3-FGFR3*	T	cg00006948	NDE	1.16	(0.86, 1.50)
					NIE	0.95	(0.81, 1.08)
					TE	1.10	(0.85, 1.38)
rs401681	5p15.33	*TERT-CLPTML*	C	cg27028750 cg26209169	NDE	1.01	(0.76, 1.33)
					NIE	1.05	(0.89, 1.25)
					TE	1.05	(0.85, 1.30)
rs2294008	8q24.3	*PSCA*	T	cg06565975 cg03405983 cg24023258 cg17888033 cg17252645	NDE	1.04	(0.78, 1.35)
					NIE	1.10	(0.90, 1.33)
					TE	1.13	(0.91, 1.38)
rs8102137	19q12	*CCNE1*	C	cg16836589 cg27475126	NDE	1.36	(1.05, 1.74)
					NIE	1.02	(0.93, 1.11)
					TE	1.38	(1.09, 1.74)

^a^Mediation models were adjusted for race/ethnicity, smoking status, pack-years of smoking, WHI arm, age at baseline, year of enrollment, follow-up time, and DNA extraction method

Abbreviations: *RA* Risk allele; *CI* Confidence interval; *NDE* Natural direct effect; *NIE* Natural indirect effect; *TE* Total effect

To address possible residual population stratification, we also conducted a sensitivity analysis restricted to white participants. This analysis led to similar results and conclusions, though rs798766 had a weaker total effect estimate (1.02 vs. 1.10), while rs401681 has a stronger total effect estimate (1.13 vs. 1.05). Interestingly, the NIE percent was lower for rs401681 (40.2%), but accounted for the entire total effect estimate for rs2294008 (over 100%).

## Discussion

Our results suggest that the effects of rs798766 and rs8102137 on bladder cancer risk are unlikely to be mediated by changes in methylation, which is consistent with the putative mechanisms underlying the carcinogenicity of these SNPs. rs798766 is in an intron of the encoding transforming, acidic coiled-coil containing protein 3 (*TACC3*) gene and has been previously linked to activating somatic mutations in nearby fibroblast growth factor receptor 3 (*FGGR3*) [14]. rs8102137 is located 6 kb upstream of the cyclin E1 gene (*CCNE1*) [15], which controls cell cycle progression to the S-phase [16]. Instead of mediation through methylation changes, rs8102137 may influence *CCNE1* splicing, since it is associated with increased expression of a *CCNE1* transcript lacking exon 7 in bladder tissue [17].

We found suggestive evidence that most of the effect of rs401681 on bladder cancer risk occurred indirectly through methylation changes at mQTL-associated CpG sites. The rs401681 SNP is in an intron of the cisplatin resistance related protein CRR9p (*CLPTM1L*) gene. The effect of rs401681 may primarily occur through an increase in methylation at cg27028750, which is not located within a known gene or CpG island. However, cg27028750 is located in a long terminal repeat element (MER-50), and genomic repeats are often targets for Polycomb repression [18].

Previous research supports a lack of association between rs401681 and differential gene expression [19, 20]. However, it is possible that rs401681 is associated with cancer-related epigenetic switching, where the repression of genes related to early development and cellular determination shifts from reversible silencing by Polycomb repressive complexes to more permanent silencing by DNA methylation [21–24]. This switch is not expected to cause changes in gene expression, but instead affects epigenetic plasticity [21]. Recent research suggests these changes predispose cells to cancer, possibly by maintaining a stem-cell-like phenotype that initiates abnormal growth and malignant transformation [24].

rs2294008 is located in the prostate stem cell antigen (*PSCA*) gene, and *PSCA* is a GPI-anchored cell surface antigen in the Ly6 family [25]. Interestingly, the rs2294008 variant has been shown to create an alternative translation start site for *PSCA* that extends the signal peptide from 11 to 20 amino acids [26]. We observed that a substantial portion of the effect of the rs2294008 T allele on bladder cancer risk may occur through methylation changes at multiple mQTL-associated CpG sites, which are annotated to four other genes in the Ly6 cluster on chromosome 8 [27]. These genes include lymphocyte antigen 6 family member K (*LY6K*), lymphocyte antigen 6 family member D (*LY6D*), Ly6/Neurotoxin 1 (*LYNX1*), and secreted Ly6/uPAR related protein 1 (*SLURP1*). *LY6K* and *LY6D* are consistently upregulated across many cancers [28] and show increased expression in bladder cancer [28]. In particular, *LY6K* has been implicated in cell growth, migration, and invasion in bladder cancer cell lines [29], and its increased expression has been associated with reduced five-year overall survival in bladder cancer patients [28, 30].

rs2294008 may also affect bladder cancer through a pathway that involves *LYNX1* and *SLURP1*. Recent research suggests that *PSCA*, *LYNX1*, and *SLURP1* bind to [31, 32] and modulate [27] the α7 subunit of nicotinic acetylcholine receptors [33, 34]. As a result, methylation-mediated changes in *LYNX1* and *SLURP1* expression might be particularly relevant among smokers by promoting the well-established connection between nicotine and bladder tumorigenesis through cascades triggered by nicotinic acetylcholine receptors that promote cell proliferation and survival [27, 35, 36].

Our study examines possible mediation through methylation for top bladder cancer genome-wide association study hits that are also mQTL. We leveraged information from previous genome-wide association studies and an existing mQTL database and used pre-diagnostic blood to explore the mechanisms underlying known associations between genetic variants and bladder cancer risk. We were able to replicate the previously reported associations between our selected genetic variants and bladder cancer (*OR*^*TE*^ estimates) [1] as well as the direction of the SNP-CpG associations reported in the ARIES mQTL database. Through this study, we also contribute new software to the research community because our approach required the adaptation of existing software to apply a multiple-mediator approach that was well described but, to our knowledge, lacked a publicly available implementation. Our sample size was not ideal for examining genetic effects, since SNPs have relatively small effect sizes. As a result, only rs8102137 had a statistically significant total effect. We were also limited by the ARIES sample size, which may not have been large enough to detect all relevant mQTL-associated mediators. Though we would expect SNPs in high linkage disequilibrium to have similar associations with mQTL-associated CpG sites and bladder cancer, we have used the top associations as proxies for the causal variants, which may have impacted our ability to detect causal effects. Despite these limitations, our results provide suggestive evidence of indirect effects through changes in DNA methylation that warrant further exploration in larger-scale studies of bladder cancer.

**Fig. 1:**
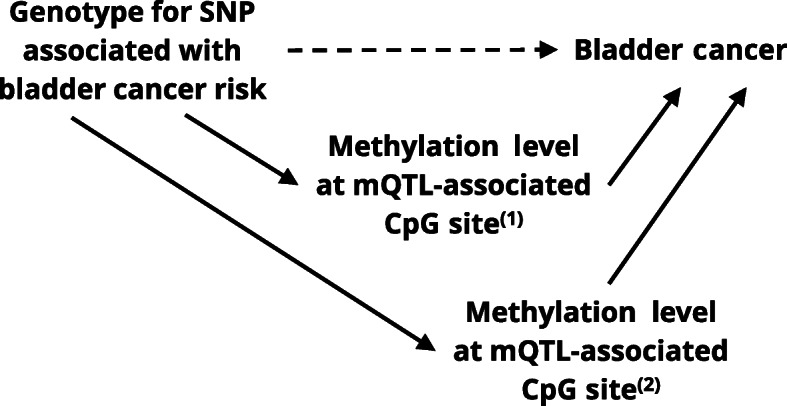
Causal directed acyclic graph shown as an illustration for a SNP with two mQTL-associated CpG site mediators, where solid arrows correspond to the indirect effect through the mediator. For this study, SNPs rs798766, rs401681, rs2294008, and rs8102137 have one to five mQTL-associated CpG site mediators

Conditional on the baseline covariates, the assumptions of mediation analyses require no-confounding of the exposure-outcome, mediator-outcome, and exposure-mediator relationships, and also require that there are no mediator-outcome confounders caused by the exposure. Analyses involving genetic variants are less prone to confounding because, aside from race/ethnicity, genotype is likely unaffected by most lifestyle factors relevant for methylation or bladder cancer. We also adjusted for a comprehensive set of covariates to address mediator-outcome confounding. We intentionally excluded cell type composition from the adjustment covariates, since it is another possible mediator that contributes to the indirect effects in our analyses. However, our analysis is limited by the accuracy of our causal model, which assumes the causal relationships depicted in Fig. 1 and may be refined in the future as more is discovered about the mechanisms underlying known associations between mQTL GWAS hits and bladder cancer.

We also assumed that there was no interaction between the mQTL SNP and its mQTL-associated mediators. We note that there are potential, though non-significant, interactions between rs798766 and cg00006948 and between rs2294008 and cg03405983 that might improve these models in more flexible approaches. However, the inclusion of an interaction term in single-mediator mediation models produced the same conclusions for rs798766 and rs2294008 in the main and relevant exploratory analyses (results not shown).

As another possible source of bias, we acknowledge error in our measurement of methylation because we only have a single measure of DNA methylation at baseline, which may imprecisely capture the methylation levels for the period that is etiologically relevant for our cases. However, the error in our measures of methylation is likely to be non-differential and is expected to attenuate the reported indirect effect estimates.

Since DNA methylation can be tissue specific, ideally, we would have conducted these analyses based on methylation data from normal bladder tissue rather than from blood. However, in a previous study of blood and brain tissue, substantial overlap was observed for mQTL from the two sites [2], indicating that at least some SNP-CpG relationships may be consistent across tissue types.

Our study suggests that a substantial proportion of the effects of rs401681 and of rs2294008 on bladder cancer risk may be mediated through methylation changes at nearby CpG sites. If confirmed by larger-scale studies, our results point to a connection between rs401681 in *CLPTM1L* and a possible nearby repression shift marked by cg27028750. A robust link between rs2294008 and risk of bladder cancer through cg24023258 may identify *LY6K* expression as a serum biomarker of bladder cancer susceptibility in non-smokers. Among smokers, further investigation of the role of rs2294008 in α7 nicotinic acetylcholine receptor signaling is warranted. Overall, this study suggests highly plausible mechanisms by which known bladder cancer risk variants are associated with susceptibility to bladder cancer and demonstrates the promising value of combining SNP and biomarker data.

## Conclusions

Though larger studies are necessary, the methylation changes associated with rs401681 and rs2294008 at mQTL-associated CpG sites may be relevant for bladder carcinogenesis, and this study demonstrates how multi-omic data can be integrated to help understand the downstream effects of genetics variants.

## Data Availability

The datasets generated and/or analyzed during the current study are available in the WHI Data and Specimen repository. The data are made publicly available upon request and according to WHI policy. The ARIES mQTL database used to identify mQTL SNPs is available online at http://www.mqtldb.org.

## Abbreviations

SNPs: Single nucleotide polymorphisms
mQTL: Methylation quantitative loci
ARIES: Accessible resource for integrated epigenomic studies
WHI: Women’s health initiative
*OR*: Odds ratio
NDE: Natural direct effect
NIE: Natural indirect effect
TE: Total effect

## References

[1] Figueroa JD, Ye Y, Siddiq A, Garcia-Closas M, Chatterjee N, Prokunina-Olsson L (2014). Genome-wide association study identifies multiple loci associated with bladder cancer risk. Hum Mol Genet.

[2] Smith AK, Kilaru V, Kocak M, Almli LM, Mercer KB, Ressler KJ (2014). Methylation quantitative trait loci (meQTLs) are consistently detected across ancestry, developmental stage, and tissue type. BMC Genomics.

[3] Wang S, Tang J, Wang M, Yuan L, Zhang Z (2010). Genetic variation in PSCA and bladder cancer susceptibility in a Chinese population. Carcinogenesis.

[4] Gaunt TR, Shihab HA, Hemani G, Min JL, Woodward G, Lyttleton O (2016). Systematic identification of genetic influences on methylation across the human life course. Genome Biol.

[5] Jordahl KM, Randolph TW, Song X, Sather CL, Tinker LF, Phipps AI, et al. Genome-wide DNA methylation in pre-diagnostic blood and bladder cancer risk in the women’s health initiative. Cancer Epidemiol Prev Biomark. 2018;27(6):689–95. 10.1158/1055-9965.EPI-17-0951 PMC5984694 29540343

[6] Hays J, Hunt JR, Hubbell FA, Anderson GL, Limacher M, Allen C (2003). The Women’s health initiative recruitment methods and results. Ann Epidemiol.

[7] Jordahl KM, Phipps AI, Randolph TW, Tindle HA, Liu S, Tinker LF (2019). Differential DNA methylation in blood as a mediator of the association between cigarette smoking and bladder cancer risk among postmenopausal women. Epigenetics.

[8] Dedeurwaerder S, Defrance M, Bizet M, Calonne E, Bontempi G, Fuks F (2014). A comprehensive overview of Infinium HumanMethylation450 data processing. Brief Bioinform.

[9] Packer BR, Yeager M, Staats B, Welch R, Crenshaw A, Kiley M (2004). SNP500Cancer: a public resource for sequence validation and assay development for genetic variation in candidate genes. Nucleic Acids Res.

[10] Sherry ST, Ward MH, Kholodov M, Baker J, Phan L, Smigielski EM (2001). dbSNP: the NCBI database of genetic variation. Nucleic Acids Res.

[11] VanderWeele T, Vansteelandt S (2014). Mediation analysis with multiple mediators. Epidemiol Methods.

[12] VanderWeele TJ, Vansteelandt S (2010). Odds ratios for mediation analysis for a dichotomous outcome. Am J Epidemiol.

[13] Valeri L, VanderWeele TJ (2013). Mediation analysis allowing for exposure–mediator interactions and causal interpretation: theoretical assumptions and implementation with SAS and SPSS macros. Psychol Methods.

[14] Kiemeney LA, Sulem P, Besenbacher S, Vermeulen SH, Sigurdsson A, Thorleifsson G (2010). A sequence variant at 4p16.3 confers susceptibility to urinary bladder cancer. Nat Genet.

[15] Kohaar I, Scott-Johnson A, Fu Y-P, Porter-Gill P, Prokunina-Olsson L (2011). Functional exploration of CCNE1 splicing forms as a possible link to bladder cancer susceptibility. Genome Biol.

[16] Siu KT, Rosner MR, Minella AC (2012). An integrated view of cyclin E function and regulation. Cell Cycle.

[17] Fu YP, Kohaar I, Tang W, Porter-Gill P, Figueroa JD, Garcia-Colsas M *et al.* Bladder cancer susceptibility variants within CCNE1 are associated with mRNA expression of an alternative splicing form. In: ASHG 2013 Annual Meeting 2013.

[18] Leeb M, Pasini D, Novatchkova M, Jaritz M, Helin K, Wutz A (2010). Polycomb complexes act redundantly to repress genomic repeats and genes. Genes Dev.

[19] Rafnar T, Sulem P, Stacey SN, Geller F, Gudmundsson J, Sigurdsson A (2009). Sequence variants at the *TERT-CLPTM1L* locus associate with many cancer types. Nat Genet.

[20] Pintarelli G, Cotroneo CE, Noci S, Dugo M, Galvan A, Carpini SD (2017). Genetic susceptibility variants for lung cancer: replication study and assessment as expression quantitative trait loci. Sci Rep.

[21] Gal-Yam EN, Egger G, Iniguez L, Holster H, Einarsson S, Zhang X (2008). Frequent switching of Polycomb repressive marks and DNA hypermethylation in the PC3 prostate cancer cell line. Proc Natl Acad Sci U S A.

[22] Jones PA, Wolkowicz MJ, Rideout WM, Gonzales FA, Marziasz CM, Coetzee GA (1990). De novo methylation of the MyoD1 CpG island during the establishment of immortal cell lines. Proc Natl Acad Sci U S A.

[23] Schlesinger Y, Straussman R, Keshet I, Farkash S, Hecht M, Zimmerman J (2007). Polycomb-mediated methylation on Lys27 of histone H3 pre-marks genes for de novo methylation in cancer. Nat Genet.

[24] Widschwendter M, Fiegl H, Egle D, Mueller-Holzner E, Spizzo G, Marth C (2007). Epigenetic stem cell signature in cancer. Nat Genet.

[25] Reiter RE, Gu Z, Watabe T, Thomas G, Szigeti K, Davis E (1998). Prostate stem cell antigen: a cell surface marker overexpressed in prostate cancer. Proc Natl Acad Sci U S A.

[26] Kohaar I, Porter-Gill P, Lenz P, Fu Y-P, Mumy A, Tang W (2013). Genetic variant as a selection marker for anti–prostate stem cell antigen immunotherapy of bladder Cancer. JNCI J Natl Cancer Inst.

[27] Fu XW, Song PF, Spindel ER (2015). Role of Lynx1 and related Ly6 proteins as modulators of cholinergic signaling in Normal and neoplastic bronchial epithelium. Int Immunopharmacol.

[28] Luo L, McGarvey P, Madhavan S, Kumar R, Gusev Y, Upadhyay G (2016). Distinct lymphocyte antigens 6 (Ly6) family members Ly6D, Ly6E, Ly6K and Ly6H drive tumorigenesis and clinical outcome. Oncotarget.

[29] de Nooij-van Dalen AG, van Dongen GAMS, Smeets SJ, Nieuwenhuis EJC, Stigter-van Walsum M, Snow GB (2003). Characterization of the human Ly-6 antigens, the newly annotated member Ly-6K included, as molecular markers for head-and-neck squamous cell carcinoma. Int J Cancer.

[30] Lee J-S, Leem S-H, Lee S-Y, Kim S-C, Park E-S, Kim S-B (2010). Expression signature of E2F1 and its associated genes predict superficial to invasive progression of bladder tumors. J Clin Oncol Off J Am Soc Clin Oncol.

[31] Ibañez-Tallon I, Miwa JM, Wang H-L, Adams NC, Crabtree GW, Sine SM (2002). Novel modulation of neuronal nicotinic acetylcholine receptors by association with the endogenous Prototoxin lynx1. Neuron.

[32] Moriwaki Y, Yoshikawa K, Fukuda H, Fujii YX, Misawa H, Kawashima K (2007). Immune system expression of SLURP-1 and SLURP-2, two endogenous nicotinic acetylcholine receptor ligands. Life Sci.

[33] Kim HS, Park WJ, Park EY, Koh JS, Hwang T-K, Kim JC (2015). Role of nicotinic acetylcholine receptor α3 and α7 subunits in detrusor Overactivity induced by partial bladder outlet obstruction in rats. Int Neurourol J.

[34] Beckel JM, Kanai A, Lee S-J, de Groat WC, Birder LA (2006). Expression of functional nicotinic acetylcholine receptors in rat urinary bladder epithelial cells. Am J Physiol Renal Physiol.

[35] Singh S, Pillai S, Chellappan S. Nicotinic acetylcholine receptor signaling in tumor growth and metastasis. J Oncol. 2011;2011. 10.1155/2011/456743. 10.1155/2011/456743 PMC3085312 21541211

[36] Chen R-J, Ho Y-S, Guo H-R, Wang Y-J (2008). Rapid activation of Stat3 and ERK1/2 by nicotine modulates cell proliferation in human bladder cancer cells. Toxicol Sci Off J Soc Toxicol.

